# The Conceptual Design of a Mechatronic System to Handle Bedridden Elderly Individuals

**DOI:** 10.3390/s16050725

**Published:** 2016-05-19

**Authors:** Silva Bruno, Machado José, Soares Filomena, Carvalho Vítor, Matos Demétrio, Bezerra Karolina

**Affiliations:** 1 R & D MEtRICs, School of Engineering, University of Minho, Guimarães 4804-533, Portugal; pg21459@alunos.uminho.pt (S.B); dmatos@ipca.pt (M.D.); karolceli@gmail.com (B.K.); 2 R & D Algoritmi, School of Engineering, University of Minho, Guimarães 4804-533, Portugal; fsoares@dei.uminho.pt (S.F.); vcarvalho@ipca.pt (C.V.); 3 Polytechnic Institute of Cávado and Ave, School of Technology, Barcelos 4750-810, Portugal

**Keywords:** mechatronic system, conceptual modelling, Ambient Assisted Living (AAL), wellbeing, Bedridden Elderly People (BEPs)

## Abstract

The ever-growing percentage of elderly people in developed countries have made Ambient Assisted Living (AAL) solutions an important subject to be explored and developed. The increase in geriatric care requests are overburdening specialized institutions that cannot cope with the demand for support. Patients are forced to have to remain at their homes encumbering the spouse or close family members with the caregiver role. This caregiver is not always physically and technically apt to assist the bedridden person with his/her meals and hygiene/bath routine. Consequently, a solution to assist caregivers in these tasks is of the utmost importance. This paper presents an approach for supporting caregivers when moving and repositioning Bedridden Elderly Peoples (BEPs) in home settings by means of a mechatronic system inspired by industrial conveyers. The proposed solution is able to insert itself underneath the patient, due to its low-profile structural properties, and retrieve and reallocate him/her. Ideally, the proposed mechatronic system aims to promote autonomy by reducing handling complexity, alter the role of the caregiver from physically handler of the BEP to an operator/supervisor role, and lessen the amount of effort expended by caregivers and BEPs alike.

## 1. Introduction

The increase in the life expectancy of elderly people in conjunction with a decline in numbers of the younger population is leading to a shift in demographics. Both MEDCs (more economically developed countries) and LEDCs (less economically developed countries) are affected by this issue, and it is becoming a global occurrence. The social changes that this issue brings have a great impact on the care provided to the elderly population. The European Commission Directorate General Health & Consumers is projecting gradual growth in the life expectancy rate in the EU. By 2025, elderly European people will represent 20% of its entire population. If this trend continues, we will see a surge in the number of people over 80 years old over the next 50 years [1,2]. This demographic trend will be accompanied by an increase in people that possess physical limitations and that thus no longer have the autonomy of movement or the ability to take care of themselves. This also means that the ratio between caregiver and elderly person will widen. The development of better solutions and techniques to help caregivers to provide care to the elderly is desperately needed if we hope to cope with these changes in demographics [3,4]. In the context of Ambient Assisted Living (AAL), few devices are available that provide the required assistance that is needed by caregivers and Bedridden Elderly Peoples (BEPs). Presently, some of the available solutions to provide support in moving and/or repositioning the elderly possess either deficiencies in their use or handling, have limited operational capabilities, or require effort from both caregivers and BEP. Moreover, the presently available home equipment solutions are, in most cases, a stripped-down, less sturdy and costly version of the hospital model. These devices usually only fulfill one purpose, and it is necessary to combine the features of several of them with physical aid from a caregiver in order to move or reposition bedridden individuals. Although many public and private organizations are aware of these issues, the development of better applications are not meeting the need. 

The main motivation behind this work is to develop a mechanism for BEPs to reacquire some autonomy of movement and thus improve their quality of life without intruding on their privacy or compromising their dignity. This paper aims to provide a design solution, in one or more aspects, to the movement and reposition issue in the household by combining different types of technologies. 

This paper is presented following this structure: Section 2 presents an approach to the issue. Further, in Section 3, a brief description on the specifications related to these sort of devices is provided, followed by some explanations on the studied design solution, explained in Section 4. Finally, the relevant conclusions are drawn, and possible future work is presented in Section 5.

## 2. An Approach to the Issue

This paper comes as a follow-up to the work previously performed in the field of the development of a mechatronic solution to assist bedridden people [5,6]. When these studies of the framework regarding solutions that provide assistance in moving and repositioning BEPs in the household were performed, a few conclusions were drawn. Initial searches for methods and systems, involving bedridden individuals, showed that many devices and contraptions already existed to aid elderly or disable individuals with their movements to, on, and from the bed. Some, such as the Sit-to-Stand (STS), developed in Italy, only approach single issues, which translates to a single function [7]. Its main purpose is to aid disabled people in standing. It greatly resembles a scissor lift and works by being positioned, without contact, under the user’s armpit, proceeding then to lift the user. Caregivers perform this activity in a similar manner, but their approach requires them to apply force. With this solution, that is no longer the case. Another device development in the academic setting is this multifunctional bed [8]. It possesses both posture changing and body transferring functionality. This device is interesting since it is very similar to regular hospital beds but also possesses a conveyer belt system as its bed frame. This enables the device to transfer patients between itself and other devices similar to it. With the configurable bed positions, this device can even adopt a configuration similar to that of a wheelchair, for example. Conferring a wheelchair form to these devices is very challenging. The Patent EP 2428197 A1 describes an approach to do so. It is described as a wheelchair that provides a lateral transfer, and its conveyer modules are driven by a tensioned wire element, pass through all modules, and are powered by a single motor [9]. A wheelchair and bed system, as is also found, had its conveyer in a configuration other than a lateral approach. The bed part of this system remained stationary, while its conveyor retrieved and deposited the patient directly from an adapted wheelchair, placed at the foot end of the bed, onto the bed [10].

The most promising devices were the ones that retrieved the BEP by inserting themselves underneath them. To avoid skin scarring or lesions, these devices used components similar to conveyor belts to add material at the same rate as they advanced forward. Two examples of such devices are the Careful Patient Mover (C-Pam) and the PowerNurse™ [11,12]. To be able to insert themselves under the BEP, these devices must possess a low profile in order not to cause discomfort during operation. These devices are designed for the hospital setting and are also independent of the base structure. They are moved from one location onto another by means of a transporting bed or gurney. Such devices also have issue aligning themselves with the patient and sometimes become misaligned during operations. Although completely independent solutions exist, they are aimed for hospital settings and are too bulky and complex for the average household. What has been deduced was that there is a need for a complete device that not only retrieves the BEP, but moves him from one location to another. Additionally, this device must be capable of self-alignment so that it does not deviate from its path. Since the operational principal of these types of applications is to insert themselves under the patient, low-profile devices are preferred. All these considerations were taken into account in the development approach of the several designs developed in the course of this project. To better compare the devices developed, a side-by-side comparison is demonstrated in Table 1. Some applications such as the transforming bed and drum motor concept have already been presented in previous papers [5,6]. The ball screw actuated conveyor concept is derived from the drum motor concept and differs in a way that, instead of reeling on a drum motor to actuate its conveyor belt, utilizes a ball screw to move a nut attached to the conveyor belt fabric. Ranked the highest in the Table 1, the low-profile center-driven conveyor presents itself as the best design to be developed. Since it is the object of study of this paper, it is explained in more detail in the following paper. 

## 3. Considered Device Specifications 

The main ISO standards, which represent the most consensus-based standardization, to follow regarding this subject are ISO 13485 and ISO 14971 [13,14]. The ISO 13485 describes the quality management system for the design and manufacture of medical devices. The adopted methodology for the development of this solution follows the VDI 2206 entitled “Design methodology for mechatronic systems”, which aims to aid in the product design process of innovative solutions that combines mechanical, electrical, and computational engineering, the base of mechatronics. For the design of this solution, only BEPs lying down (decubitus) were considered. They could either be lying on their backs (dorsal decubitus), lying on one side (lateral decubitus), or lying on their stomachs (ventral decubitus). When moving bedridden people, it is preferred, for stability and safety, to move them in the dorsal decubitus. From the data that show the percentile distribution of height in the American population (from 2007–2008), it can be observed that beds that are 1981 mm (6.5 feet) long will cover over 99% of the population [15]. Regarding the width, anthropometric data estimates for Swedish and English adults indicate that the shoulder breadth for 95% of the male populations is fewer than 510 mm [16,17]. Since the device being developed is for a home setting, the dimensions of the doors must be taken into account. In accordance with DIN 18100 (Doors; wall openings for doors with dimensions in accordance with DIN 4172) and ISO/TC 162, standardized wall openings for home settings usually possess a minimum width of 625 mm. However, Italian accessibility regulations state that internal doors must have at least 800 mm of usable clear width, while Scottish Building Standards state that this value must be at least 775 mm where a door is approached head-on [18]. The Portuguese law of accessibility indicates that an inside door must possess a clear width no fewer than 770 mm [19]. To follow the appointed regulations and in order to clear the door, the device must possess a width fewer than 770 mm while considering clearance. The values from the percentile distribution of weight in the American population (from 2007–2008) were used to define the maximum load. These values provide data on the percentile distribution of the mass under the value, separated by age and sex, of the US population. Its analysis indicates that, if the maximum mass specification is set to 155 kg (approximately 340 lb), the designed device may accommodate 98.8% of the population [20,21].

**Figure 1:**
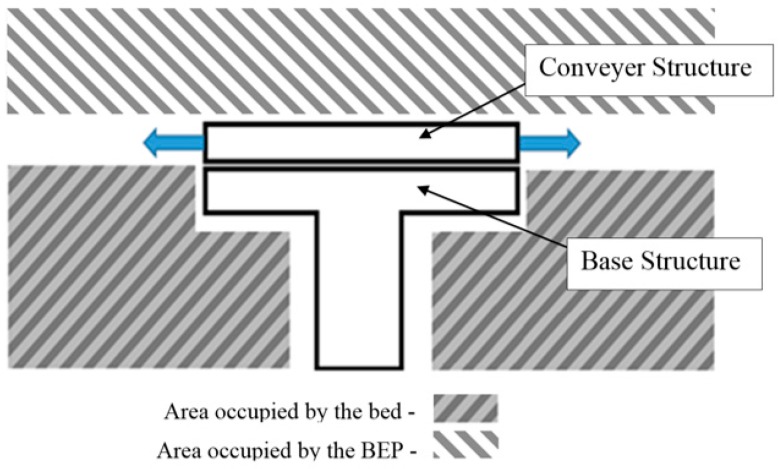
Obstructed areas.

The areas marked in Figure 1 represent the obstructed space in which no device(s) can be placed. Placing the drive components in any place rather than inside the conveyor structure only increases complexity and mechanical constrains. The side of the conveyor structure is also not adequate for the placement of the device(s) that will drive the conveyor belt, since these locations will possess a revolving edge on which the belt will rotate. The need for this edge stems from the necessity of providing a tip on which to facilitate the insertion of the conveyor structure under the patient. The design solution to this problem was first to divide the conveyor structure in three modules, with each module having an independent drive. The intent within this approach was to divide the workload among the three modules. These modules would possess two center motors opposite to each other. Since the workload is distributed, this measure aids in reducing the required torque, the size of the required motor, and consequently its overall footprint.

Utilizing the data in Table 2 [22], a distribution of the mass that will affect each section can be further studied. Accordingly, the upper section covers the head, neck, arms, and thorax and must handle up to about 55 kg of maximum load. The middle section covers the forearms, hands, abdomen, pelvis, and thighs and must handle up to about 82 kg of maximum load. The lower section covers the legs and feet, and must handle up to about 50 kg of maximum load. 

The final specifications for the development of this application, as well as a brief justification on the principles behind these values, are shown in Table 3.

## 4. Proposed Solution

**Figure 2:**
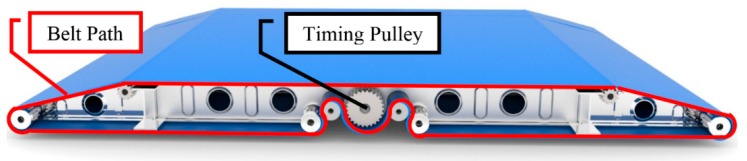
Timing belt path.

The proposed solution involves three conveyor modules. Each individual module drives a conveyer by means of two centered motors opposed to each other. The motors will drive two timing belts that are physically attached to the conveyor belt. The three modules are supported by telescopic rails that extend to both sides of the device, enabling bed-to-bed transfer. The extension and retraction of the telescopic rails, and consequently the conveyor modules, is powered by a simple crossed tensioned cabled system explained further below. Figure 2 shows a sectioned view of the inner workings of the conveyor belt modules. In this figure, the timing belt path is shown by the continuous line. This better illustrates the inner workings and how the conveyor belt and the timing belt set wraps around both timing and tensioning pulleys.

**Figure 3:**
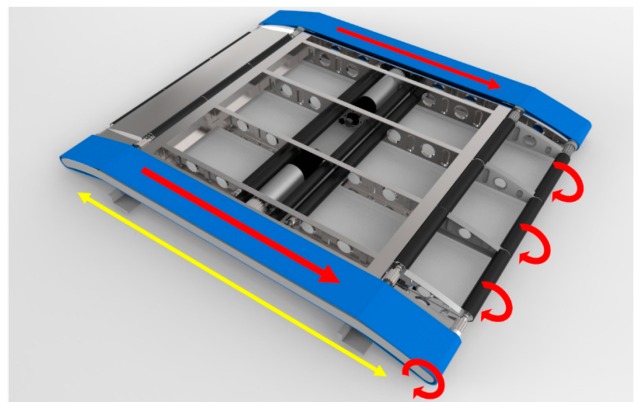
Inner workings and power transmission.

The pulleys, marked by circular arrows, as rotating in Figure 3, are connected to a shaft that runs the length of the conveyor module. One of these shafts exits on both sides of the conveyor module. As the centered conveyor belt is driven by the center motors, this belt confers motions to these shafts. These shafts drive two smaller tensioned belts on each side. The path of these belts describe a smaller profile then the centered conveyor belt in order to create the opening shown in Figure 3 by the yellow bidirectional arrow. The purpose of this opening is to provide an attachment location to the telescopic rail and a seamless joint between modules so that the break in belt material is negligible.

### 4.1. Custom Drive Mechanism Using Crossed Tensioned Elements

**Figure 4:**
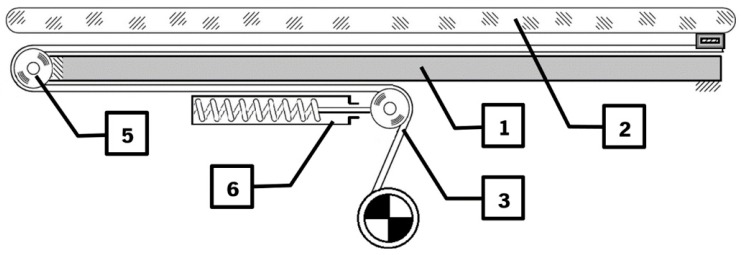
Track layout.

The need for a custom drive mechanism stemmed from an issue regarding a movement system that needed to provide a stroke in both directions, without having any part of the structure obstructing either the patient or any other elements. The operational procedure to retrieve a patient is to align the device with the bed. The device moves in the direction of the bed until the base is under the bed, and the conveyor modules are over the bed. Lower the conveyor modules so that its base is flushed with the bed mattress and advance the conveyor structure supported by the telescopic rails. In the operation described, little to no space is available between the device and the bed, and the traditional mechanisms, such as Scotch yoke and the modern linear actuators, could not be employed without infringing on the areas motioned. These mechanisms possess limitations in either of range, direction or mechanical constrains. To solve this issue, a custom drive mechanism using crossed tensioned elements was developed. This mechanism is shown in Figure 4 and it is composed of a base (1), a trolley (2), a flexible element (belt, wire, cord, chain, *etc.*) (3), rails (4), bearings/pulley (5), tensioners (6), spools (7), a motor (8), gearbox (9), and shaft (10). The mechanism is quite simple; as the motor raps the tensioned element, the trolley is pulled in a track in the direction of the pulley (5). If another similar track is connected in an opposite configuration, its tensioned element wraps around in a counter fashion to the first track; as one track is pulled, the other is winded up, and *vice versa*.

**Figure 5:**
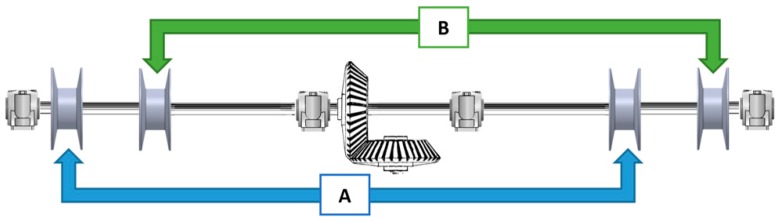
Shaft layout.

Figure 5 shows an example with the proposed number of pairs of tracks (two) to aid in stability. All spools are fixed to the same shaft, and motion is transmitted to this shaft by means of a single motor. Each spool in pair “α” has its counterpart (group “A”) in pair “β” (group “B”).

This device is placed under the conveyor structure attached to a telescopic pillar. Since the telescopic rails extend on both sides, a mechanical limitation is required. This mechanism naturally provides a maximum stroke from its tensioned element that is less than the stroke of the telescopic rails. A small working prototype of this movement mechanism was built at 1:3 scale in order to test the viability for the operational purpose. The test performed showed that the mechanism behaves as expected, thereby the movement requirements were achieved as expected.

**Figure 6:**
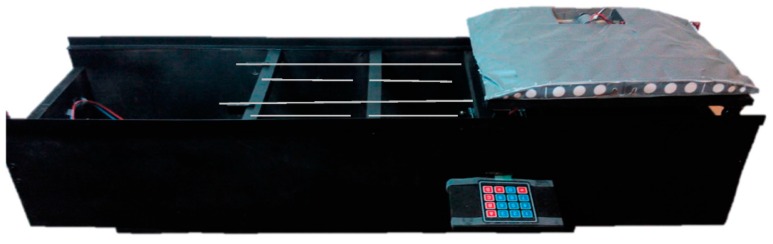
Tensioned elements mechanism prototype.

In order to demonstrate the operations performed by this mechanism, a prototype at 1:3 scale was built. This also served as a proof of concept for a future build. The construction and components are very rudimentary and easily available. The trolley discussed in the previous chapter is in fact the conveyer module and is represented here by the grey model of the conveyer structure. When the system is put into effect, the conveyer structure is shifted alongside the rails that simulate the extended telescopic rails. Figure 6 shows the conveyer shifted to the right and the tension element (cord) configurations in that position.

**Figure 7:**
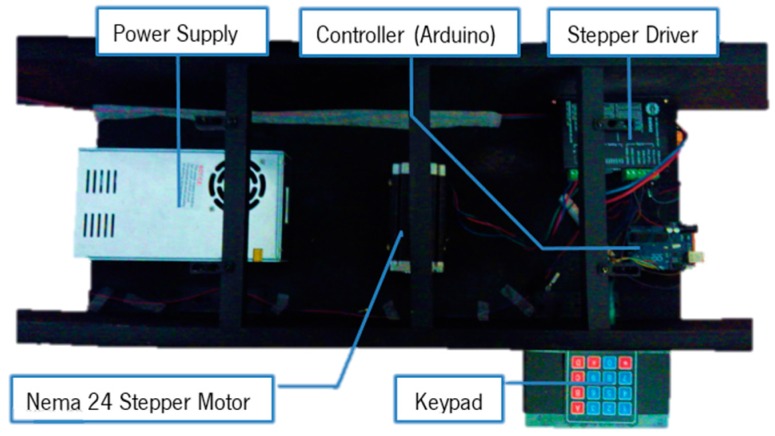
Tensioned elements mechanism prototype and electronic components.

The electronic components for this prototype are shown in Figure 7. The controller sends the movement information to the stepper driver that controls a stepper motor. The stepper drive serves another function, which is the power management from the power supply, since the tension value present in the Arduino board is not adequate to drive the motor. User input and motion control is provided by a numerical keypad. 

With this prototype a proof of concept was made. The configurations of the electronics only serve to boost the simplicity of the mechanism and reduce component management from the controller device. 

### 4.2. Controller Device

**Figure 8:**
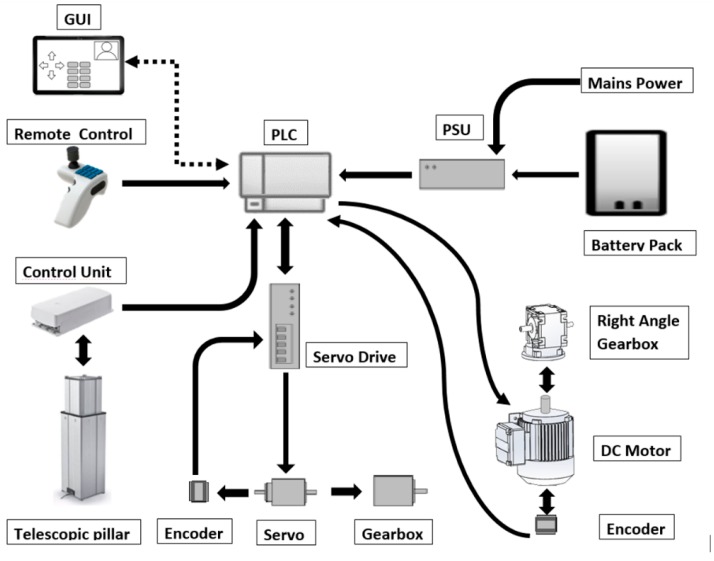
Component connections diagram.

Medical equipment needs to stand up to conditions that are not expected of normal devices. These devices functions must provide added insurance that they will not fail since the user’s well-being may be at stake. To do so, they must be designed to handle issues such as corrosive substances such as bodily fluids and EMF (Electromagnetic Fields). The control system that is sold in conjunction with the actuators and other equipment designed for healthcare tend to be enclosed regarding its inner workings. Other devices are more open to development such as microcontrollers or microprocessor boards, but on their own they are sensible to corrosion, impact, and EMF. For an automatic operation of the proposed solution, the PLC (Programmable Logic Controller) is best suited for this sort of application since these devices are submitted to extremely hazardous conditions. Figure 8 shows the elements that interact with the PLC. The PLC acts as the controller for these applications but receives its commands via a remote control. This remote possesses inputs for all the necessary commands as well as a three-axis joystick to operate the mecanum wheels located at the base of the solution. These wheels and the conveyor servo motors are controlled through the PLC by a servo drive that handles the servo motor operations and the feedback from the encoders. The Telescopic pillar that adjust the height of the Conveyor structures is controlled trough the PLC via its own control unit. Power can either be provided through the battery pack or directly by a mains outlet. The motor with a right angle gearbox is used to drive the custom movement system by means of a crossed tensioned element. The control of the device can either be performed by a GUI (Graphical User Interface) on a mobile device or by the developed ergonomic control remote. Figure 8 shows the relations between the components and the connections between them.

The several automated processes require feedback in order to assure proper operations. The device must possess means to obtain information from its environment and the actors involved so that protocols in the PLC can be implemented to properly protect the patients that utilizes this applications.

#### 4.2.1. Encoder

The encoder is a crucial feedback sensor to ensure that the required movements are performed and, if not, return a value to correct this error. Thanks to this device and through some logic operations, speed and position can be obtained. These devices are to be coupled to the motors that either drive the *mecanum* wheels or the conveyor belt system. In the case of the latter, special considerations must be taken regarding both the size of the encoder and its ability to operate in a corrosive environment, all the while taking into account that it is performing near a live human being. This last statement is true for most of the sensors involved in this type of application. Some of these devices do have an ingress protection rating, but these do not contemplate interaction with live human bodies.

#### 4.2.2. Load Presence Detection

At this point, the proposed design uses single strips of force sensing resistors to determinate whether the patient is on the bed. In this design, these force sensing resistors, or simply FSRs, are positioned in a layout so that the body sections that apply most of the pressure may be registered. In the upper body section, these are the head and upper back; in the middle section, the pelvis; in the lower section, the calves and the heels. After retrieving the patient the sensor is aware of the presence of a “load” and this enables more information to implement more automation conditions. In situations such as the disappearance of this load, without the patient deposit operation being performed, enables the implementation of additional safety features safeguard the user’s well-being. Ideally, single strips of FSRs would not be utilized; instead, a matrix of several FSR cells would create a mesh of sensors that could not only measure but also locate where pressure is applied.

#### 4.2.3. Bed Detection Sensor

The detection of the presence of the bed under the device, to transfer the BEP from or to it, may be comprised of a combination of one or several sets of photo resistors, photodiodes, or simple mechanical switch sensors. Inductive sensors are not considered due to their operating nature and to the lack of such elements for their proper function. In addition to detecting the presence of the bed, these sensors can be utilized to determine whether the device is completely extended or whether it is in alignment with the base structure. 

#### 4.2.4. User Interface

**Figure 9:**
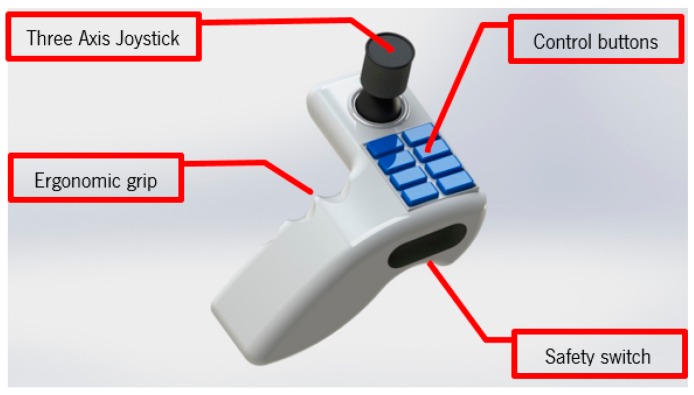
Ergonomic remote with three-axis joystick.

Figure 9 shows the designed ergonomic controller developed for this application, which combines the controls found on hospital bed remotes and a multi-axis joystick found in the robotics industry for the control of the mecanum wheel, since these require a control of three degrees of freedom. The three-axis joystick enables the omnidirectional control of the mecanum wheels in the base structure, while the control buttons each control one of the operations of the transfer system. The ergonomic shape of the controller enables the user to hold the controller in its left hands with ease. No operation occurs if the user does not press the safety switch that enables the other control options. 

**Figure 10:**
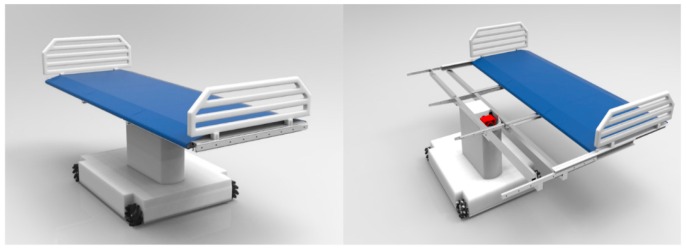
3D rendering of the complete solution.

A rendering of the overall solution is presented in Figure 10. All the mentioned components are assembled in this configuration. The final profile of the conveyor structure is 47 mm, achieving the objective of a low-profile solution. Additionally, it is worth mentioning that no components are encumbering the space over or under the conveyor modules.

## 5. Conclusions

The emerging problem of handling BEPs is undeniable and further development of new devices and applications for improving the quality of life for these individuals is imperative. In this paper, a conceptual design solution to this problem was presented. One prototype, for validating the accuracy of the movement in both directions, has been developed. This prototype demonstrated the use of the full stroke of the telescopic rail guides while providing a low profile and reduced footprint. The present design is intended for transference of a patient between bed and bed since the form that it provides is also that of a bed. Nevertheless, it possesses the design foundation to provide an update to a device that could transform from a bed and a wheelchair. Such a conversion would enable transference of the patient from bed to bed, chair to bed, or chair to chair. Now, the next step consists in the fabrication of a second prototype for validating the structural behavior and safety of the patient. However, since the system will handle live individuals, some issues emerge regarding legal authorizations for using live test subjects, which hinder the advances of a second prototype. Not only these legal regulations but also other standards, some already mentioned here, dictate how certain aspects of how the device must be built. Where some standards oppose further advances by what they stipulate, a lack of regulations on other components contribute as much for the delays in development. For instance, some devices that possess an IP rating code and an ingress protection rating do not clarify as to their use with human beings. Authors are trying to approach Portuguese institutions as partners to solve some of those issues.

## Figures and Tables

**Table 1:** Decision matrix.

Criteria	Weight	Transforming Bed	Drum Motor Driven Conveyor	Ball Screw Actuated Conveyor	Low-Profile Center-Driven Conveyor
**Safety**	2	4	2	6	8
**Comfort**	1.75	7	1	6	7
**Robustness**	1.5	6	4	7	8
**Construction Complexity**	1.25	3	7	6	7
**Handling Complexity**	1.25	3	4	8	8
**Programing Complexity**	1.25	3	7	6	7
**Cost**	1	4	5	5	6
**Raw Score**	-	31	30	44	51
**Weighted Score**	-	32.25	37.5	52.5	61.5
**Rank**	-	4	3	2	1

**Table 2:** Human body mass distribution.

Body Segment	Relative Mass	Individual Member Mass (kg)	Combined Mass (kg)
**Head and neck**	8.1%	12.6	12.6
**Arm**	5.6%	4.3	8.7
**Forearm**	3.2%	2.5	5
**Hand**	1.2%	0.9	1.9
**Thorax**	21.6%	33.5	33.5
**Abdomen**	13.9%	21.5	21.5
**Pelvis**	14.2%	22	22
**Thigh**	20%	15.5	31
**Leg**	9.3%	7.2	14.4
**Foot**	2.9%	2.2	4.5
		**Total mass:**	155

**Table 3:** Device specifications.

Device Specification		Justification	Value	Unit
Length		99th percentile population height	1981	mm
Width	Minimum	95th percentile shoulder width	510	
	Maximum	Accessibility standards	770	
Maximum Load	Upper section	Mass impact of the body segments that are exerting force onto each section	54.715	kg
	Middle section		81.375	
	Lower section		49.910	
	Total	99th percentile population mass	155	
